# Gene regulatory elements determine efficacy of BCMA-targeted CAR-T cell products

**DOI:** 10.1136/jitc-2026-016839

**Published:** 2026-09-18

**Authors:** Viktoria Blumenberg, Kristen B June, Filippo Birocchi, Giulia Escobar, Charlotte Graham, Yueyang Fan, Merle K Phillips, Sangwoo Park, Deshea Harris, Henry Anderson, Amanda A Bouffard, Christopher Kelly, Magdi Elsallab, Trisha R Berger, Kathleen ME Gallagher, Mark B Leick, Adele Mucci, Marcela V Maus

**Affiliations:** 1 Cellular Immunotherapy Program, Mass General Brigham Cancer Institute, Boston, Massachusetts, USA; 2 Harvard Medical School, Boston, Massachusetts, USA; 3 Comprehensive Cancer Center, King’s College London, London, UK

**Keywords:** Chimeric antigen receptor - CAR, Adoptive cell therapy - ACT, Multiple Myeloma

## Abstract

In relapsed/refractory multiple myeloma, the approved B-cell maturation antigen (BCMA)-targeted chimeric antigen receptor (CAR)-T cell product ciltacabtagene autoleucel (cilta-cel) shows higher response rates than idecabtagene vicleucel (ide-cel) in pivotal trials and real-world comparisons. This advantage has been attributed to the biparatopic dual-VHH (dVHH) binder of cilta-cel. However, the products also differ in the gene regulatory elements driving CAR expression: cilta-cel uses a human elongation factor α (EF1α) promoter in conjunction with a woodchuck hepatitis post-transcriptional regulatory element (EF1α^+WPRE^), whereas ide-cel uses a myeloproliferative sarcoma virus enhancer (MND) promoter without WPRE (MMD^-WPRE^). The role of these elements in CAR-T cell efficacy remains unexplored.

We generated lentiviral vectors where the CAR transgene was composed of either a dVHH-based or a single-chain variable fragment (scFv)-based BCMA-CAR with a 4-1BB costimulatory and a CD3ζ signaling domain, expressed either by EF1α^+WPRE^ or MND^-WPRE^. Primary human T cells or a nuclear factor of activated T cells (NFAT)-GFP Jurkat reporter line were compared at matched vector copy numbers (VCN) or CAR surface expression. Antitumor efficacy was assessed in MM.1S-engrafted NSG mice.

When transduced at the same multiplicities of infection, MND^-WPRE^ CAR-T cells reached higher VCN and transduction rates than EF1α^+WPRE^ regardless of the binder type. In an NFAT-GFP reporter assay, unstimulated MND^-WPRE^-driven cells showed higher NFAT and CD69 expression, consistent with greater tonic signaling compared with EF1α^+WPRE^. In vivo, independently of the BCMA binder moiety, EF1α^+WPRE^-driven CAR-T cells demonstrated superior cytokine secretion and tumor control along with greater survival of mice treated with EF1α^+WPRE^compared with MND^-WPRE^-driven CAR-T cells.

CAR-T cells expressing the CAR under MND^-WPRE^ showed greater gene transfer during manufacturing, but this did not translate into superior function in vivo. The advantage attributed to cilta-cel’s dual VHH binder was lost when expressed under MND^-WPRE^ and, conversely, ide-cel’s scFv binder achieved better antitumor activity when expressed under EF1α^+WPRE^ than under MND^-WPRE^. The efficacy difference between our CAR constructs in xenograft models therefore tracked with the choice of regulatory elements driving CAR expression independently of the binder. It may be better to choose gene regulatory elements based on the CAR-T cell function they confer rather than on expression strength alone.

## Introduction

 The B-cell maturation antigen (BCMA)-targeted chimeric antigen receptor (CAR)-T cells ciltacabtagene autoleucel (cilta-cel) and idecabtagene vicleucel (ide-cel) have achieved remarkable success in relapsed/refractory multiple myeloma. In terms of antitumor efficacy, cilta-cel has outperformed ide-cel, in real-world comparisons (progression-free survival; p<0.001, HR=0.45, CI 0.36 to 0.63)^1^ as well as their respective pivotal trials (overall response rate of 97% for cilta-cel vs 73% for ide-cel).^2 3^ Cilta-cel is the only Food and Drug Administration (FDA)-approved CAR-T cell product that uses a nanobody-based binder, which engages two BCMA epitopes; all others use a single-chain variable fragment (scFv)-based binder. Therefore, its superior efficacy has been attributed to the biparatopic binder design, a strategy which has since been pursued for solid tumors.^4^

However, cilta-cel and ide-cel also differ in a second, overlooked variable: the gene regulatory elements used to drive CAR transgene expression. The cilta-cel CAR transgene is driven by an elongation factor α (EF1α) promoter stabilized with a woodchuck hepatitis post-transcriptional regulatory element (WPRE). EF1α is a ubiquitously expressed mammalian cellular promoter and a strong constitutive driver of transgene expression that is used in the majority of clinically approved lentiviral CAR-T cell products.^5–8^ The WPRE is a commonly used element in lentiviral vectors, located at the 3′-untranslated region of the transgene to increase messenger RNA (mRNA) levels by enhancing transcript termination, which ultimately enhances transgene expression.^9^ The ide-cel transgene is expressed under the myeloproliferative sarcoma virus enhancer (MND) promoter without WPRE.^10^ MND is a gamma retrovirus-derived promoter, thus absent in human cells, and drives high transgene expression with high per copy activity.^5 11^ Ide-cel is the only FDA-approved lentiviral CAR-T cell product expressing the CAR under the MND promoter.

The level and kinetics of CAR expression throughout antigen exposure are increasingly recognized as determinants of CAR-T cell function.^12^ Gene regulatory elements determine the transcriptional and post-transcriptional control of CAR expression next to posttranslational mechanisms such as internalization, degradation, and recycling of CAR proteins.^13^ Therefore, we asked whether the divergent regulatory configurations driving CAR expression in cilta-cel and ide-cel, rather than their different binder platforms to which their efficacy has been attributed, account for their different clinical outcomes. Based on the clinical efficacy data, we hypothesized that the EF1α^+WPRE^ configuration confers superior efficacy over MND^-WPRE^, for both dual-VHH (dVHH) and scFv binders. To test this, we reconstructed the binder and the regulatory configuration of cilta-cel and ide-cel from their published sequences and expressed each binder under both configurations while holding all other elements identical. We do acknowledge that we are using reconstructed versions of the molecular constructs and are not producing bona fide “ide-cel” or “cilta-cel” products in our experimental approach, but the nomenclature is used for simplicity to describe the transgene designs.

## Methods

### Cell lines

SUP-T1 (CRL-1942), Jurkat (TIB-152), Hek293T (CRL-3216) and MM.1S (CRL-2974) cell lines were obtained from the American Type Culture Collection, STR-authenticated and *Mycoplasma* tested. Cells were cultured in RPMI medium (Thermo Fisher Scientific #72400047) supplemented with 10% fetal bovine serum (Life Technologies, #A52567-01) and 1% penicillin/streptomycin (Thermo Fisher Scientific #15140163), referred to as R10, at 37°C, 5% CO₂. The MM.1S cell line was transduced to stably express a click beetle green luciferase-GFP reporter and GFP-sorted for bioluminescence imaging (BLI). A nuclear factor of activated T cells (NFAT)-GFP Jurkat reporter line was generated by lentiviral transduction with the NFAT eGFP Reporter Lentivirus (BPS Bioscience, cat #79922), and selected for transduced cells through puromycin.

### Mice

NSG mice (Jackson #JAX:005557) were bred at the MGB Cancer Institute. Experiments followed an Institutional Animal Care and Use Committee (IACUC)-approved protocol #2020N000114. Each in vivo experiment comprised five groups: untransduced T cells (UTD) and each binder (scFv, dVHH) under each configuration (MND^-WPRE^, EF1α^+WPRE^) with n=5 mice per group (25 mice per experiment). Mice at 8–12 weeks were intravenously injected with 1×10^6^ MM.1S across all in vivo experiments. Only mice with confirmed tumor engraftment were randomized by BLI tumor burden. CAR-T cells were thawed, rested one hour in R10 with 20 IU/mL interleukin (IL)-2 (PeproTech #200-02) and injected intravenously (curative dose 2×10^6^ cells, stress dose 0.5×10^6^ cells). Weekly BLI assessments were performed through intraperitoneal injection of D-luciferin by two veterinary technicians blinded to treatment (Thermo Fisher Scientific #88294, 30 mg/mL), imaged with an Ami HT/HTX spectral apparatus and analyzed in Aura V.5.0.0.

### Production of lentiviral vectors

Amino acid sequences were codon-optimized for human expression, synthesized (GenScript), and cloned into a third-generation self-inactivating lentiviral vector backbone. Lentiviral particles were produced by transient transfection of HEK293T cells (Lipofectamine 3000; transfer plus pMDLg/pRRE, pRSV-Rev, pVSV-G packaging plasmids; details in online supplemental methods), concentrated by ultracentrifugation, and stored at -80°C. Vector titers were determined on SUP-T1 cells.

### Titration of lentiviral vectors on primary T cells

Leukapheresis products from anonymous healthy human donors (MGH blood bank, IRB-exempt) were used to isolate T cells (Stemcell #15061). T cells were thawed, activated with CD3/CD28 Dynabeads (Life Technologies #40 203D; 3:1 bead-to-T-cell ratio) and plated at 1×10^5^ cells/100 µl. On day one, T cells were transduced with the lentivirus across a range of multiplicities of infection (MOI). UTD from matching donors served as controls. Cultures were expanded with fresh R10/IL-2 every two days, with Dynabead removal on day 5, until day 14. mCherry or CAR surface expression was measured by flow cytometry (Miltenyi #130-136-457) on day 14 (gating is described in online supplemental figure S1).

### Digital droplet PCR of CAR transgene copies

For each construct, three MOIs yielding 30% transduction (±10%) were selected, and genomic DNA was purified from the corresponding bulk (unsorted) populations (5×10^5^ cells), including UTDs (DNeasy Blood and Tissue Kit, Qiagen #69506; Qubit High Sensitivity DNA Assay, Thermo Fisher Scientific #Q33230). Droplet generation, amplification and reading were performed on Bio-Rad instruments (Automated Droplet Generator, C1000 Touch, QX600). No-template and matched-donor UTD controls were included in every run (see methods in online supplemental appendix). One MOI per construct was chosen so that bulk vector copy number (VCN) was closely matched across all four constructs.

### CAR-T cell production

For functional and in vivo studies, T cells were activated as above (1×10⁶/mL, 24-well) and transduced at the selected MOI titrated to match bulk VCN across constructs. At day 8, CAR+ cells were enriched by BCMA-PE labeling (Miltenyi #130-133-888 #130-048-801) and magnetic column separation (Miltenyi #130-042-401; purity >95% positivity for mCherry and CAR surface expression) and cultured in IL-2 until day 14. CAR-T cells were cryopreserved in FBS+10% DMSO (Thermo Fisher Scientific #85190; see methods in online supplemental appendix).

### NFAT reporter assay

Tonic signaling was assessed in the NFAT-GFP Jurkat reporter cell line, transduced across MOI 0.625–7.5. To compare tonic signaling independently of CAR surface expression level, EF1α^+WPRE^ and MND^-WPRE^ conditions were matched for CAR surface mean fluorescent intensity (BCMA-PE). On day 7 after transduction, in the absence of BCMA^+^ target cells, NFAT-GFP and CD69 expression were quantified by flow cytometry.

### Flow cytometry

Cells were stained 30 min at 4°C, resuspended in 4′,6-diamidino-2-phenylindole (DAPI; Miltenyi #130-111-570, 1:100) for live/dead cell discrimination immediately prior to analysis on a NovoCyte Penteon (Agilent). CAR expression was detected using recombinant human BCMA-PE (Miltenyi #130-133-888) or BCMA-APC (Miltenyi #130-136-457). CD69 was detected with an anti-CD69 antibody (clone FN50, BioLegend #310912). Data were analyzed in FlowJo V.10.10.0 (gating is described in online supplemental figure S1).

### Cytokine analysis

Plasma from tumor-bearing mice (EDTA) was assayed for human cytokines by a bead-based multiplex immunoassay (LEGENDplex Human CD8/NK Panel, BioLegend #741187). Samples were diluted 1:10 in duplicates, acquired on Penteon (Agilent) and concentrations (pg/mL) interpolated from a standard curve using LEGENDplex analysis software.

## Results

### CAR-T cells driven by MND^-WPRE^ show higher transduction than EF1α^+WPRE^ at matched MOI across both BCMA binders

**Figure 1:**
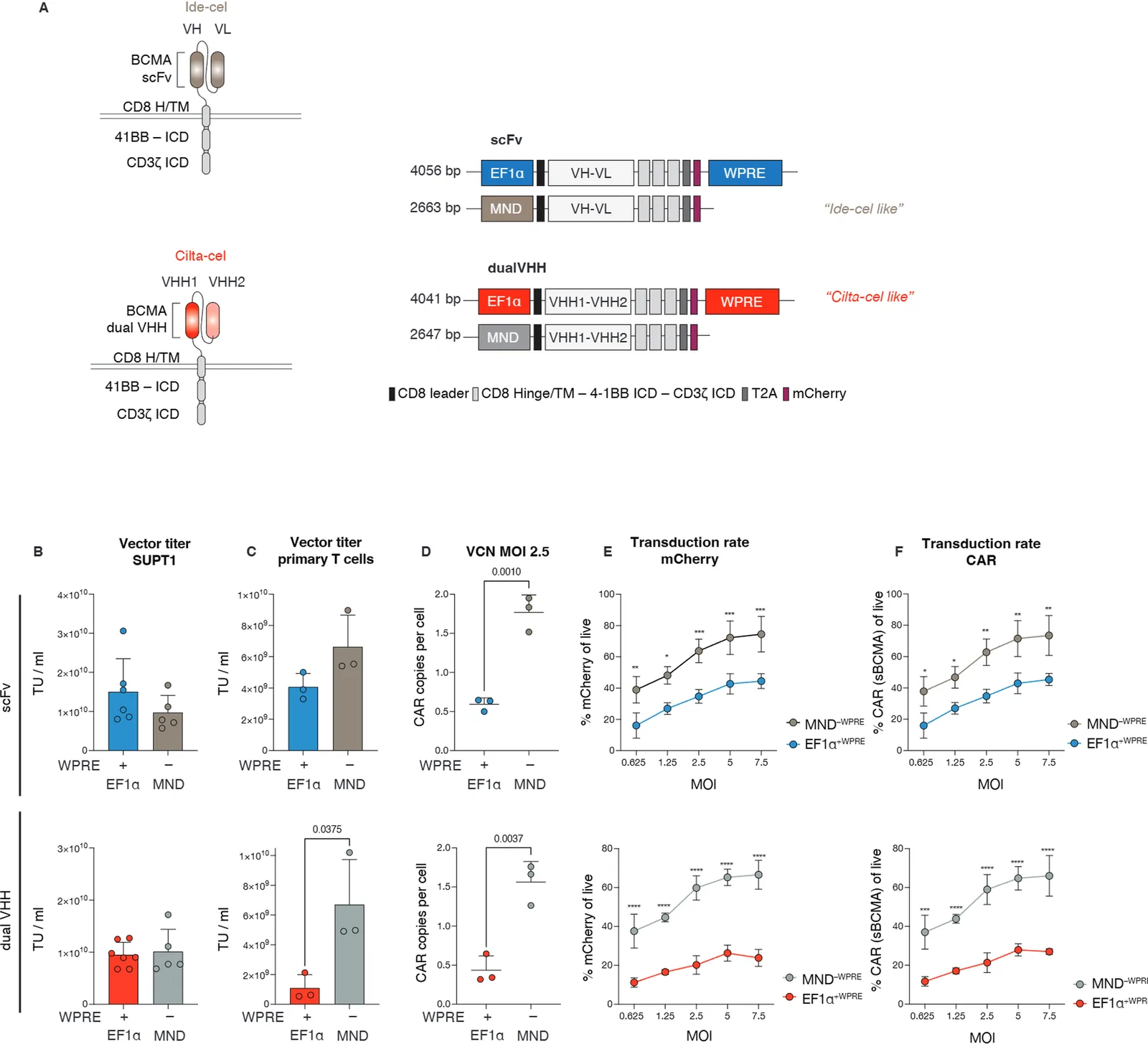
CAR-T cells driven by MND^-WPRE^ show higher transduction than EF1α^+WPRE^ at matched MOI across BCMA binders. (**A**) Schematic of the four BCMA CAR constructs. An ide-cel-like scFv and a cilta-cel-like biparatopic dual VHH were each cloned into two regulatory configurations (EF1α^+WPRE^ and MND^-WPRE^), yielding four constructs. (**B**) Lentivirus was produced in HEK293T cells, titrated on SUP-T1 cells to define MOI. Functional lentiviral titer (TU/mL) on SUP-T1 cells. (**C**) Functional lentiviral titer (TU/mL) on primary human T cells. (**D**) VCN (CAR copies per cell, across the whole unsorted population, including untransduced cells) at matched MOI 2.5 by ddPCR. (**E**) Transduction rate as %mCherry^+^ of live cells across MOI 0.625–7.5. (**F**) Transduction rate as %CAR^+^ (BCMA-APC) of live cells across MOI 0.625–7.5. scFv (top) and dual VHH (bottom); EF1α^+WPRE^ compared with MND^-WPRE^ in each panel. Data are mean±SEM of n=3 biological replicates from three independent human T-cell donors. Statistical analysis: (**C–D**) unpaired two-tailed t-test; (**E–F**) two-way ANOVA with Sidak’s correction for multiple comparisons. *p<0.05, **p<0.01, ***p<0.001, ****p<0.0001. ANOVA, analysis of variance; BCMA, B-cell maturation antigen; bp, base pairs; CAR, chimeric antigen receptor; cilta-cel, ciltacabtagene autoleucel; ddPCR, digital droplet PCR; EF1α, elongation factor α; ICD, intracellular domain; ide-cel, idecabtagene vicleucel; MND, myeloproliferative sarcoma virus enhancer; MOI, multiplicity of infection; ns, not significant; scFv, single-chain variable fragment; TM, transmembrane domain; TU, transducing units; VCN, vector copy number; WPRE, woodchuck hepatitis virus post-transcriptional regulatory element.

To dissect the contributions of the gene regulatory configuration (MND^-WPRE^ vs EF1α^+WPRE^) and binder architecture (an ide-cel-like scFv vs a cilta-cel-like biparatopic dVHH), we expressed each binder under the two clinically used regulatory configurations. All constructs shared identical transmembrane and intracellular architecture (a CD8 hinge and transmembrane domain, a 4–1BB costimulatory domain, and a CD3ζ intracellular domain) linked to mCherry via a T2A ribosomal skip element. We did not recapitulate their full, proprietary manufacturing and therefore refer to them as “ide-cel-like” and “cilta-cel-like” (figure 1A). Following lentiviral vector production in HEK293T cells, vectors were titrated on SUP-T1 cells by transduction rate to define MOIs, and primary human T cells were transduced across an MOI series. Functional titers did not differ between regulatory configurations on SUP-T1 cells (figure 1B). On primary T cells, those transduced with the dVHH vector reached lower transduction efficiencies with the EF1α^+WPRE^ cassette than with the MND^-WPRE^ (p=0.0375, figure 1C), with a similar trend for the scFv constructs. At a matched MOI of 2.5, CAR-T cells expressing either binder under MND^-WPRE^ had higher VCN than those expressing it under EF1α^+WPRE^ (p=0.0010 and p=0.0037 for scFv and dVHH, respectively; figure 1D). Consistent with this, CAR-T cells driven by MND^-WPRE^ showed higher transduction than those driven by EF1α^+WPRE^ at matched MOI, measured by mCherry (figure 1E, online supplemental figure S1) and CAR surface expression (figure 1F) for both binders.

### CAR-T cells driven by MND^-WPRE^ show greater tonic signaling than EF1α^+WPRE^ across both BCMA binders

**Figure 2:**
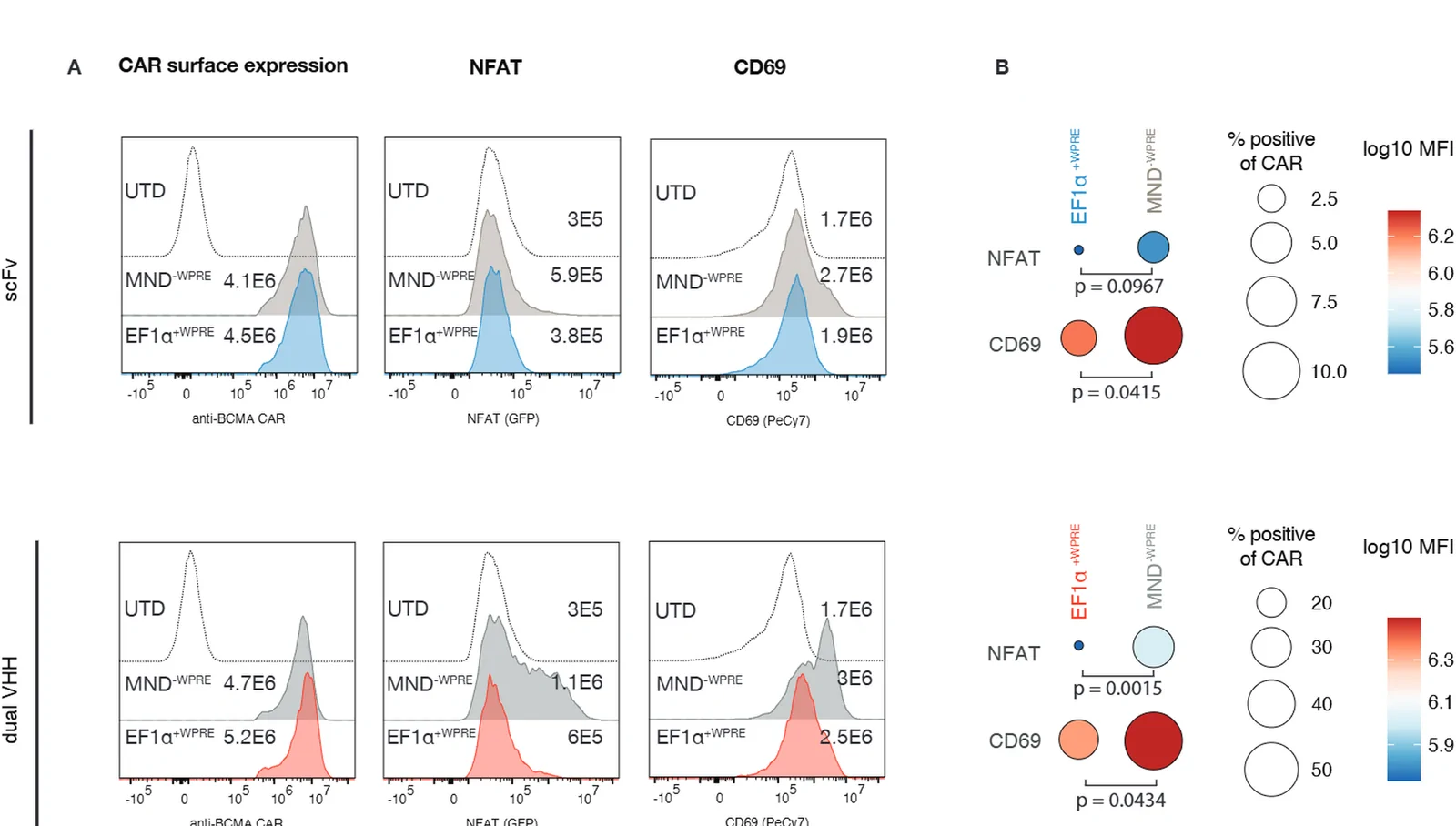
CAR-T cells driven by MND^-WPRE^ show greater tonic signaling than EF1α^+WPRE^ across BCMA-binders. Tonic signaling assessed in NFAT-GFP Jurkat reporter cells in the absence of BCMA^+^ target cells, at matched CAR surface expression. (**A**) Representative histograms of CAR surface expression (assessed through BCMA-PE), NFAT-GFP, and CD69 for EF1α^+WPRE^ versus MND^-WPRE^, with geometric MFI annotated. (**B**) Bubble plot of NFAT and CD69 in the CAR^+^ population, where bubble size denotes percentage positive and color denotes log_10_ MFI. scFv (top) and dual VHH (bottom) shown throughout. Data from n=3 independent experiments using the NFAT-GFP Jurkat reporter cell line. scFv versus dVHH: EF1α^+WPRE^, NFAT p=0.003, CD69 p=0.0016; MND^-WPRE^, NFAT p=0.0003, CD69 p=0.0046. P values of unpaired t-tests of % positive are shown. BCMA, B-cell maturation antigen; CAR, chimeric antigen receptor; EF1α, elongation factor α; MFI, mean fluorescent intensity; MND, myeloproliferative sarcoma virus enhancer; NFAT, nuclear factor of activated T cells; scFv, single-chain variable fragment; UTD, untransduced; WPRE, woodchuck hepatitis virus post-transcriptional regulatory element.

To assess CAR-T cell state in the absence of target antigen, we expressed the vectors in an NFAT-GFP Jurkat reporter cell line. At matched CAR surface expression (online supplemental figure S2), CAR-T cells driven by MND^-WPRE^ showed higher NFAT-GFP and CD69 expression than those driven by EF1α^+WPRE^, with overall greater tonic signaling for the dVHH than the scFv constructs (figure 2A and B).

### CAR-T cells driven by EF1α^+WPRE^ confer superior in vivo tumor control and survival than MND^-WPRE^ across both BCMA binders

**Figure 3:**
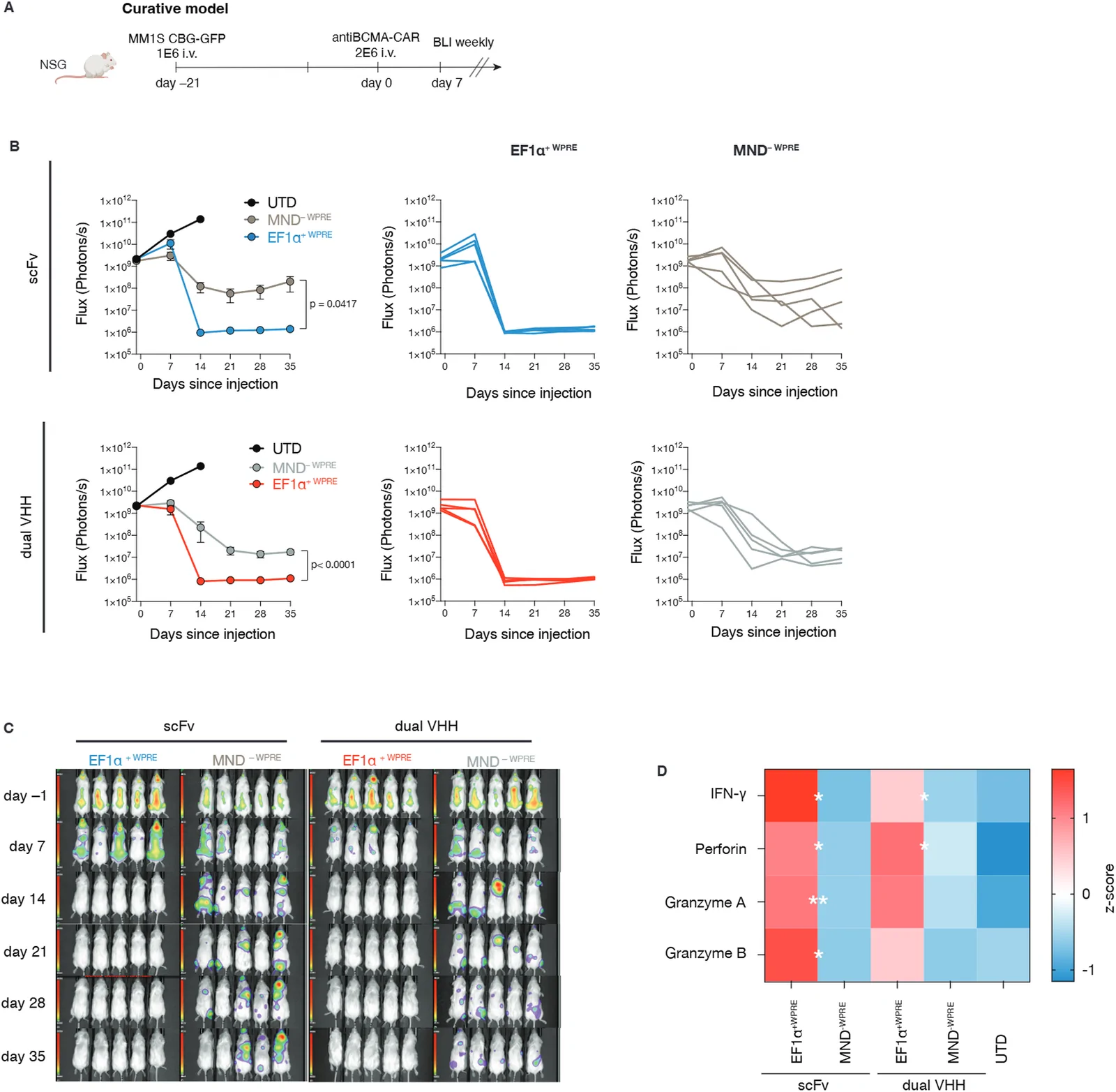
CAR-T cells driven by EF1α^+WPRE^ confer superior in vivo tumor control and effector cytokine secretion across BCMA binders at a curative dose. (**A**) Curative MM.1S xenograft model: NSG mice received 1×10⁶ MM.1S CBG-GFP i.v. on day -21 and 2×10⁶ CAR-T cells (unsorted) i.v. on day 0, with weekly BLI from day 7. (**B**) Tumor burden (total flux, photons/s) over time for UTD, EF1α^+WPRE^, and MND^-WPRE^ (left, group mean); individual EF1α^+WPRE^ (middle) and MND^-WPRE^ (right) trajectories. Data are mean±SEM; n=5 mice per group. The same UTD control group (n=5) is shown in both the scFv and dVHH panels for reference. (**C**) Representative BLI images from day -1 to day 35 for EF1α^+WPRE^ and MND^-WPRE^. Baseline images at day -1 show not randomized groups. (**D**) Heatmap of plasma cytokines (z-score) quantified by multiplex immunoassay on day 7. scFv (top) and dual VHH (bottom). Data are mean±SEM; n=5 mice per group. Statistical analysis: (**B**) unpaired two-tailed t-test on log-transformed total flux performed at the last time point at which all mice were alive; (**D**) unpaired two-tailed t-test comparing five mice per group at day 7. *p<0.05, **p<0.01, ***p<0.001, ****p<0.0001. BCMA, B-cell maturation antigen; BLI, bioluminescence imaging; CAR, chimeric antigen receptor; CBG, click beetle green; EF1α, elongation factor α; IFN-γ, interferon-gamma; i.v., intravenous; MND, myeloproliferative sarcoma virus enhancer; scFv, single-chain variable fragment; UTD, untransduced; WPRE, woodchuck hepatitis virus post-transcriptional regulatory element.

**Figure 4:**
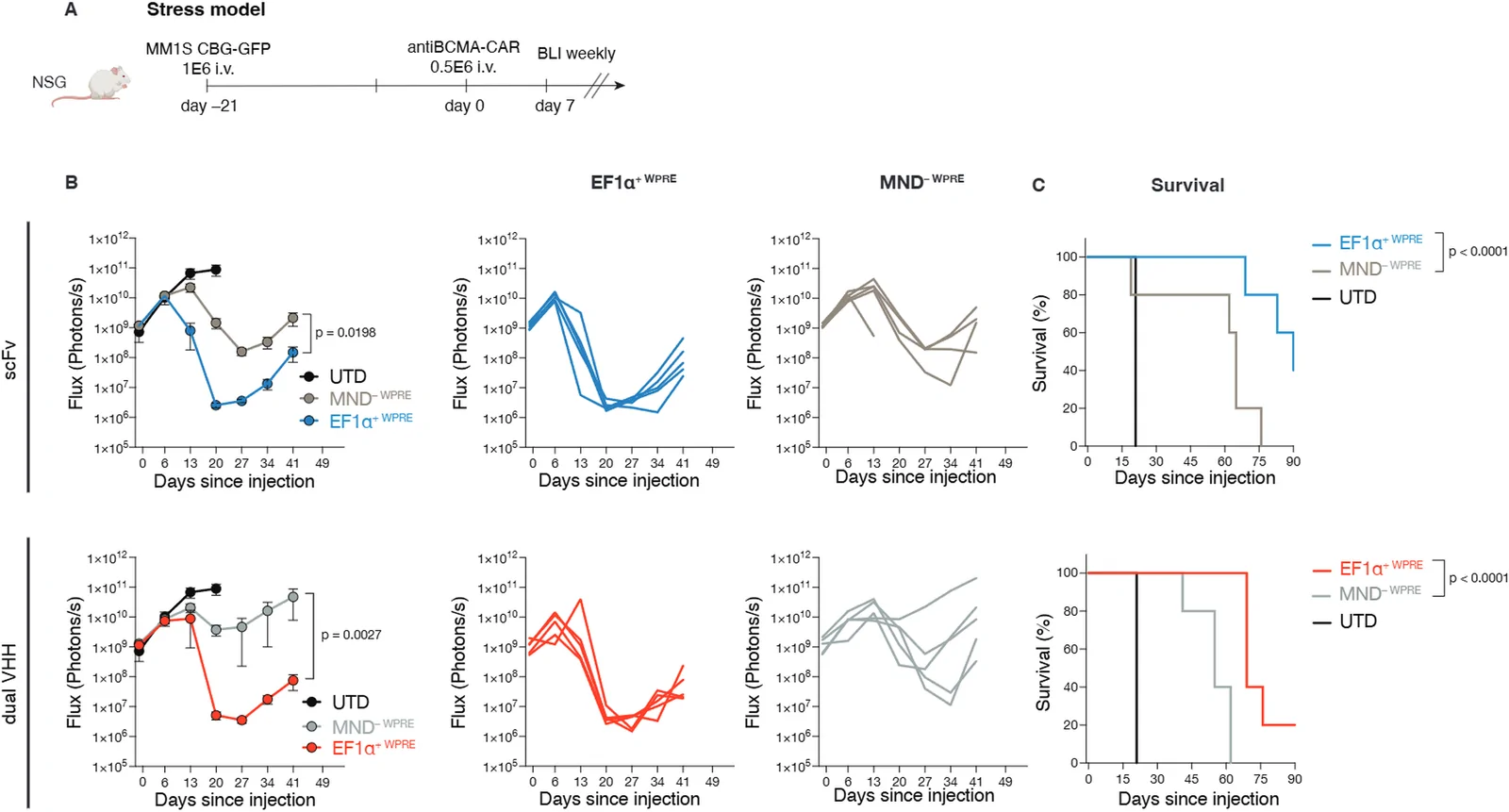
CAR-T cells driven by EF1α^+WPRE^ confer superior in vivo tumor control and survival across BCMA binders at a stress dose. (**A**) Stress-dose MM.1S xenograft model: NSG mice received 1×10⁶ MM.1S CBG-GFP i.v. on day -21 and 0.5×10⁶ CAR-T cells i.v. (enriched for CAR+) on day 0, with weekly BLI. (**B**) Tumor burden (total flux, photons/s) over time for UTD, EF1α^+WPRE^, and MND^-WPRE^ (left, group mean); individual EF1α^+WPRE^ (middle-left) and MND^-WPRE^ (middle-right) trajectories. The same UTD control group (n=5) is shown in both the scFv and dVHH panels for reference. (**C**) Kaplan-Meier overall survival. Data are mean±SEM; n=5 mice per group. Statistical analysis: (**B**) unpaired two-tailed t-test on log-transformed total flux performed at the last time point at which all mice were alive; (**C**) survival by log-rank (Mantel-Cox) test. BCMA, B-cell maturation antigen; BLI, bioluminescence imaging; CAR, chimeric antigen receptor; CBG, click beetle green; EF1α, elongation factor α; i.v., intravenous; MND, myeloproliferative sarcoma virus enhancer; scFv, single-chain variable fragment; UTD, untransduced; WPRE, woodchuck hepatitis virus post-transcriptional regulatory element.

To study the impact of the regulatory configuration on CAR-T cell function in primary human T cells without bias from differential integration, we titrated the vectors to matched VCN by ddPCR (online supplemental figure S3A). The titration yielded narrow VCN ranges for both binders (0.5–0.9 for scFv and dVHH; online supplemental figure S3B) with comparable frequencies of mCherry^+^ (30–49% for scFv, 20–47% for dVHH; online supplemental figure S3C) and CAR^+^ (30–43% for scFv, 23–47% for dVHH; online supplemental figure S3D) cells. We then treated MM.1S-bearing mice with a curative dose of 2×10⁶ CAR-T cells (figure 3A). By BLI, CAR-T cells driven by EF1α^+WPRE^ cleared tumor across both binder platforms, whereas MND^-WPRE^-driven CAR-T cells did not (figure 3B and C). Consistent with this, mice treated with EF1α^+WPRE^-driven CAR-T cells had higher plasma levels of effector cytokines, such as interferon-γ, perforin, granzyme A and B, at day seven than those treated with MND^-WPRE^-driven CAR-T cells (figure 3D). For the stress model of MM.1S-bearing mice, the CAR-T cell population was further enriched for the CAR+ fraction through column-based sorting with narrow VCN ranges in the CAR^+^-enriched population (1.3–2.3 for scFv, 1.4–1.9 for dVHH; online supplemental figure S4A-C). To increase stringency, the dose was lowered to 0.5×10⁶ CAR-T cells (figure 4A). Again, CAR-T cells driven by EF1α^+WPRE^ outperformed those driven by MND^-WPRE^, regardless of the binder (figure 4B). This translated into prolonged survival of mice treated with EF1α^+WPRE^-driven CAR-T cells compared with mice treated with MND^-WPRE^ across both binders (figure 4C).

## Discussion

In reconstructed CAR vectors modeling the two clinically approved BCMA-targeted products ide-cel (scFv expressed under MND^-WPRE^) and cilta-cel (dVHH expressed under EF1α^+WPRE^) the regulatory configuration determined antitumor efficacy independently of the binder, and may therefore contribute to the efficacy difference between ide-cel and cilta-cel. We observed that CAR-T cells driven by MND^-WPRE^ showed higher transduction rates and greater tonic signaling than CAR-T cells driven by EF1α^+WPRE^. Furthermore, MND^-WPRE^-driven CAR-T cells showed inferior effector function independent of the antigen-binding domain. Thus, the superior efficacy of the cilta-cel-like construct is abrogated when its biparatopic binder is expressed under MND^-WPRE^, and the ide-cel-like scFv achieved greater antitumor activity under EF1α^+WPRE^ than it did not reach under MND^-WPRE^. Because functional comparisons in vivo were performed at matched VCN and proportion of CAR positive cells, differences in outcome cannot be explained by differences in vector integration or transduction rate. To our knowledge, this is the first evidence that the regulatory configuration can determine antitumor efficacy independently of the binder design and may therefore contribute to the differences between the two approved BCMA-targeted products, which has so far been attributed to cilta-cel’s biparatopic binder.

Our in vitro findings are consistent with previous reports that MND drives higher titers^14^ and transduction rates^14 15^ than EF1α in scFv-based CD19 CAR-T cells. These metrics capture how efficiently a vector can be manufactured, but not necessarily how well CAR-T cells perform.

Others have shown that a high density of CAR molecules promotes receptor clustering, tonic signaling and inferior antitumor effects.^16^ Accordingly, in BCMA-targeted scFv CAR-T cells, high CAR surface density has been associated with reduced cytotoxicity in vitro and in vivo and with transcriptomic and epigenetic signatures of tonic signaling and exhaustion.^17^ Furthermore, transient rest from CAR signaling can prevent or reverse exhaustion and restore the antitumor functionality.^18^ These mechanisms cannot explain the greater tonic signaling of the MND^-WPRE^ compared with EF1α^+WPRE^ constructs we observed here, as CAR surface density was matched between regulatory element configurations in the NFAT reporter cell line, and the CAR itself (hinge, transmembrane and costimulatory domains) was identical. CAR surface density is a steady-state measure, set by the relation of synthesis, internalization and degradation, whereas NFAT and CD69 signaling accumulates over time. In addition to steady-state CAR density, as assessed here, the temporal behavior of CAR expression might matter as well. Consistent with a potential value of lower or dynamically regulated CAR expression, Eyquem *et al* placed the CAR under control of the endogenous T cell receptor constant alpha chain locus (rather than a constitutive promoter such as EF1α), which lowered CAR levels on target binding, reduced exhaustion and improved efficacy compared with EF1α-driven CAR-T cells.^19^ Similarly, expression of an scFv-based CD19 4-1BB CAR under the Wiskott-Aldrich syndrome promoter in lentiviral vectors reproduced TCR-like expression kinetics, although tumor control did not differ from EF1α-driven CAR-T cells in a Burkitt lymphoma model.^20^ Whether gene regulatory elements permit such modulation depends on its responsiveness to activation-associated transcription factors, and synthetic promoters built from defined transcription factor binding sites can be engineered to couple CAR expression to T-cell state.^21^

Our in vivo findings appear to contradict studies in CD19-targeted scFv-based CAR-T cells, where MND-driven CAR-T cells outperformed EF1α at borderline significance in a Raji model^14^ and more clearly in a Nalm-6 model.^15^ This may reflect a CAR-T cell manufacturing bias, as neither study reports normalization to VCN, so that differences in transduction may have confounded the comparison. Alternatively, the differences could be target antigen related, since CAR-T cell activation has been shown to be antigen density-dependent.^22 23^ CD19 is expressed at high, homogeneous density on B-lineage blasts,^24^ whereas BCMA is expressed at lower, more heterogeneous density and is subject to shedding.^25^ Thus, regulatory configurations may be differentially favorable by antigen level, where MND-driven CAR-T cells might be better suited to higher, and EF1α^+WPRE^-driven cells to lower target antigen expression levels. Given this discrepancy between MND-driven CARs when targeting CD19 versus BCMA, it remains unclear how these gene regulatory elements affect CAR-T cell responses in solid tumors, where antigen density might be more heterogeneous and CAR-T cells face chronic antigen exposure and a suppressive tumor microenvironment.^4^ Furthermore, all constructs used here contain a 4-1BB domain and costimulatory domains differ in their downstream signaling cascades with CD28 driving greater signaling per receptor molecule. Thus the effect for 4-1BB CARs observed here cannot be assumed to hold for CD28-based products.^16^

Our study has several limitations: We compared the two clinically deployed configurations as units and did not isolate the contribution of the individual elements; the promoter effect (MND vs EF1α), the presence of WPRE and the difference in vector length. Each of these factors may contribute to the efficacy, such as WPRE increases transgene mRNA and expression,^6^ and their roles in the observed differences remain to be defined. As tonic signaling was assessed only in an NFAT-GFP Jurkat reporter line as a surrogate readout but not in primary human CAR T-cells, we cannot establish a causal link between the greater tonic signaling of MND^-WPRE^ and its inferior in vivo efficacy. In addition, xenograft models were performed with one tumor cell line, so it remains unclear whether the superiority of EF1α^+WPRE^ is maintained at different antigen densities. Furthermore, xenograft models in immunodeficient mice do not capture CAR-T cell persistence and memory formation, which are established drivers of durable treatment responses in patients, and the distinct contribution from the biparatopic binding design compared with the scFv remains unclear. Finally, we reconstructed the molecular design of the clinically approved constructs but not their proprietary manufacturing processes.

Our results raise the possibility that the inferior efficacy of ide-cel compared with cilta-cel in pivotal trials and real-world data may be attributable, in part, to the choice of MND^-WPRE^ rather than EF1α^+WPRE^ for CAR transgene expression, although this remains to be confirmed for the clinical products. Regulatory elements are selected based on metrics used to benchmark vectors such as titer, transduction, and VCN and may be chosen deliberately to maximize CAR transgene expression. However, this might favor the configuration that promotes inferior effector function. Although MND^-WPRE^ gave higher titers, transduction rates and VCN, it conferred inferior tumor control in vivo at matched VCN. Therefore, regulatory elements may be better selected by the antitumor efficacy they confer rather than manufacturing parameters alone.

## Supplementary material

10.1136/jitc-2026-016839 online supplemental file 1

## Data Availability

Data are available upon reasonable request.
