# Supplementary material for: Gene regulatory elements determine efficacy of BCMA-targeted CAR-T cell products
Source: J Immunother Cancer. 2026 Sep 18;14(9):e016839. doi: 10.1136/jitc-2026-016839 (PMC13599996; doi:10.1136/jitc-2026-016839)
Supplement: Supplementary file 1 [file jitc-14-9-s001.pdf]

SUPPLEMENTARY APPENDIX

Gene regulatory elements determine efficacy of BCMA-targeted CAR-T cell products

Viktoria Blumenberg[1,2], Kristen B. June[1], Filippo Birocchi[1,2], Giulia Escobar[1,2], Charlotte Graham[3], Yueyang Fan[1,2], Merle K. Philipps[1,2], Sangwoo Park[1,2], Deshea Harris[1], Henry Anderson,[1] Amanda Bouffard[1], Christopher Kelly[1], Magdi Elsallab[1,2], Trisha R. Berger[1], Kathleen M. E. Gallagher[1,2], Mark B. Leick[1,2], Adele Mucci[1] and Marcela V. Maus[1,2]

[1] Cellular Immunotherapy Program, Mass General Brigham Cancer Institute, Boston, MA, USA
[2] Harvard Medical School, Boston, MA, USA
[3] Current address: Comprehensive Cancer Centre, King's College London, London, UK.

SUPPLEMENTARY METHODS

Study design

This study was designed to determine whether configuration of regulatory elements or the antigen-binding domain are the primary determinants of efficacy of clinically approved BCMA-targeted CAR-T cells. Each binder was expressed under the two configurations used in the approved products: the cilta-cel configuration (EF1α^{+WPRE}) and the ide-cel configuration (MND^{-WPRE}) yielding four constructs. Functional comparisons in primary human T cells were performed at matched VCN, or at matched CAR surface expression for tonic-signaling assays. *In vivo* efficacy was assessed in an MM.1S xenograft model in NSG mice. Group size was informed by prior studies using this model, in which differences of the observed magnitude in tumor burden and survival were detectable with n=5 per group. A formal a priori power calculation was not performed. Tumor inoculation, CAR-T cell injection, bioluminescence measurements and health monitoring were performed on all groups in the same sessions to avoid batch- and order-related

confounders. Given the small group sizes (n=5), distributional assumptions were not formally tested.

<u>Production of lentiviral vectors</u>

The scFv (ide-cel-like) antigen-binding domain corresponds to the murine anti-BCMA 11D5-3 clone (patent US20200261501A1; VL, SEQ ID NO 7; VH, SEQ ID NO 8; 218 linker, SEQ ID NO 22). Full binder sequence:

GACATTGTGCTCACTCAGTCACCTCCCAGCCTGGCCATGAGCCTGGGAAAAAGGGCCACCATCTCCTGTAGAGCCAGTGAGTCCGTCACAATCTTGGGGAGCCATCTTATTCACTGGTATCAGCAGAAGCCCGGGCAGCCTCCAACCCTTCTTATTCAGCTCGCGTCAAACGTCCAGACGGGTGTACCTGCCAGATTTTCTGGTAGCGGGTCCCGCACTGATTTTACACTGACCATAGATCCAGTGGAAGAAGACGATGTGGCCGTGTATTATTGTCTGCAGAGCAGAACGATTCCTCGCACATTTGGTGGGGGTACTAAGCTGGAGATTAAGGGAAGCACGTCCGGCTCAGGGAAGCCGGGCTCCGGCGAGGGAAGCACGAAGGGGCAAATTCAGCTGGTCCAGAGCGGACCTGAGCTGAAAAAACCCGGCGAGACTGTTAAGATCAGTTGTAAAGCATCTGGCTATACCTTCACCGACTACAGCATAAATTGGGTGAAACGGGCCCCTGGAAAGGGCCTCAAATGGATGGGTTGGATCAATACCGAAACTAGGGAGCCTGCTTATGCATATGACTTCCGCGGGAGATTCGCCTTTTCACTCGAGACATCTGCCTCTACTGCTTACCTCCAAATAAACAACCTCAAGTATGAAGATACAGCCACTTACTTTTGCGCCCTCGACTATAGTTACGCCATGGACTACTGGGGACAGGGAACCTCCGTTACCGTCAGTTCCGCGGCCGCA

The biparatopic dual-VHH (cilta-cel-like) binder corresponds to LCAR-B38M (patent US20210128618A1; VHH1, SEQ ID NO 1 and 2; VHH2, SEQ ID NO 3 and 4; Inter-VHH linker, SEQ ID NO 5 and 16). Full binder sequence:

CAGGTCAAACTGGAAGAATCTGGCGGAGGCCTGGTGCAGGCAGGACGGAGCCTGCGCCTGAGCTGCGCAGCATCCGAGCACACCTTCAGCTCCCACGTGATGGGCTGGTTTCGGCAGG

CCCCAGGCAAGGAGAGAGAGAGCGTGGCCGTGATCGGCTGGAGGGACATCTCCACATCTTACGCCGATTCCGTGAAGGGCCGGTTCACCATCAGCCGGGACAACGCCAAGAAGACACTGTATCTGCAGATGAACAGCCTGAAGCCCGAGGACACCGCCGTGTACTATTGCGCAGCAAGGAGAATCGACGCAGCAGACTTTGATTCCTGGGGCCAGGGCACCCAGGTGACAGTGTCTAGCGGAGGAGGAGGATCTGAGGTGCAGCTGGTGGAGAGCGGAGGCGGCCTGGTGCAGGCCGGAGGCTCTCTGAGGCTGAGCTGTGCAGCATCCGGAAGAACCTTCACAATGGGCTGGTTTAGGCAGGCACCAGGAAAGGAGAGGGAGTTCGTGGCAGCAATCAGCCTGTCCCCTACCCTGGCCTACTATGCCGAGAGCGTGAAGGGCAGGTTTACCATCTCCCGCGATAACGCCAAGAATACAGTGGTGCTGCAGATGAACTCCCTGAAACCTGAGGACACAGCCCTGTACTATTGTGCCGCCGATCGGAAGAGCGTGATGAGCATTAGACCAGACTATTGGGGGCAGGGAACACAGGTGACCGTGAGCAGCACTAGT

Replication-deficient lentivirus was produced by transfecting plasmids into HEK293T cells cultured in DMEM supplemented with GlutaMAX and HEPES (Thermo Fisher Scientific, cat. #72400047), 10% FBS (Life Technologies cat. #A52567-01), 1% penicillin and streptomycin (Thermo Fisher Scientific cat. #15140163 and cat. #15140163). HEK293T cells were plated at 3×106 cells/plate in a 150mm cell culture plate. After 3 days, HEK293T cells at 80% confluence were transfected using Lipofectamine 3000 (Invitrogen, cat. #L3000075) with the CAR transfer plasmid (15µg) together with the packaging plasmids pMDLg/pRRE (gag-pol; 18 µg), pRSV-REV (Rev; 18 µg), and pVSV-G (VSV glycoprotein envelope; 7 µg) per 150 mm plate. Supernatant was collected and filtered (0.45µm, Millipore Sigma, cat. # S2HVU02RE) 72 h after transfection. Filtered virus was concentrated by ultracentrifugation on the Thermo Fisher Scientific Sorvall WX+ Ultracentrifuge (2h, 25 000 RPM at 4°C) and stored at −80 °C. SUP-T1 cells were transduced with serial dilutions of vector, and the transduction rate was measured as the percentage of mCherry$^+$ cells by flow cytometry three days after transduction. Functional titer (transducing units per mL, TU/mL) was calculated from dilutions within the linear range

(transduction rate below ~30%, where one integration event per cell can be assumed) as: TU/mL = (number of cells transduced × fraction mCherry$^+$ × dilution factor) / volume of vector. Multiplicity of infection (MOI) for subsequent transductions was defined as TU per target cell based on this titer.

<u>Digital droplet PCR of CAR transgene copies</u>

Digital droplet PCR (ddPCR) was performed using using a custom assay specific for the CD3ζ and 4-1BB domain in the CAR transgene (Bio-Rad, forward sequence: GTCGGAAGAAGCTGCTGTA, reverse sequence: GAATTTCACGCGCAGTTCG, probe sequence: AGAGGAGGAGGAAGGCGGCT for FAM) and the reference gene RPP30 (Bio-Rad, Assay ID: dHsaCP1000485 for HEX).

<u>Statistical analysis</u>

Statistical analyses were performed in GraphPad Prism v11.0.1 (GraphPad Software). For the MOI titration the mCherry$^+$ and CAR transduction rate was compared by repeated-measures two-way ANOVA. Pairwise comparison at each MOI used Šidák's multiple comparisons test. Vector copy number at matched MOI was compared between promoters by unpaired two-tailed t test. *In vivo* tumor burden was quantified as total flux (photons/s) by bioluminescence imaging. At the last imaging time point at which all animals in the compared groups remained alive, log2-transformed flux was compared between vectors by unpaired two-tailed t test. Overall survival was estimated by the Kaplan-Meier method 90 days after CAR-T cell injection and compared between groups by the log-rank (Mantel-Cox) test. Data are presented as mean ± SEM. P values below 0.05 were considered statistically significant; exact P values are reported in the figures where feasible. n for each panel is defined in the corresponding figure legend.

SUPPLEMENTARY FIGURES

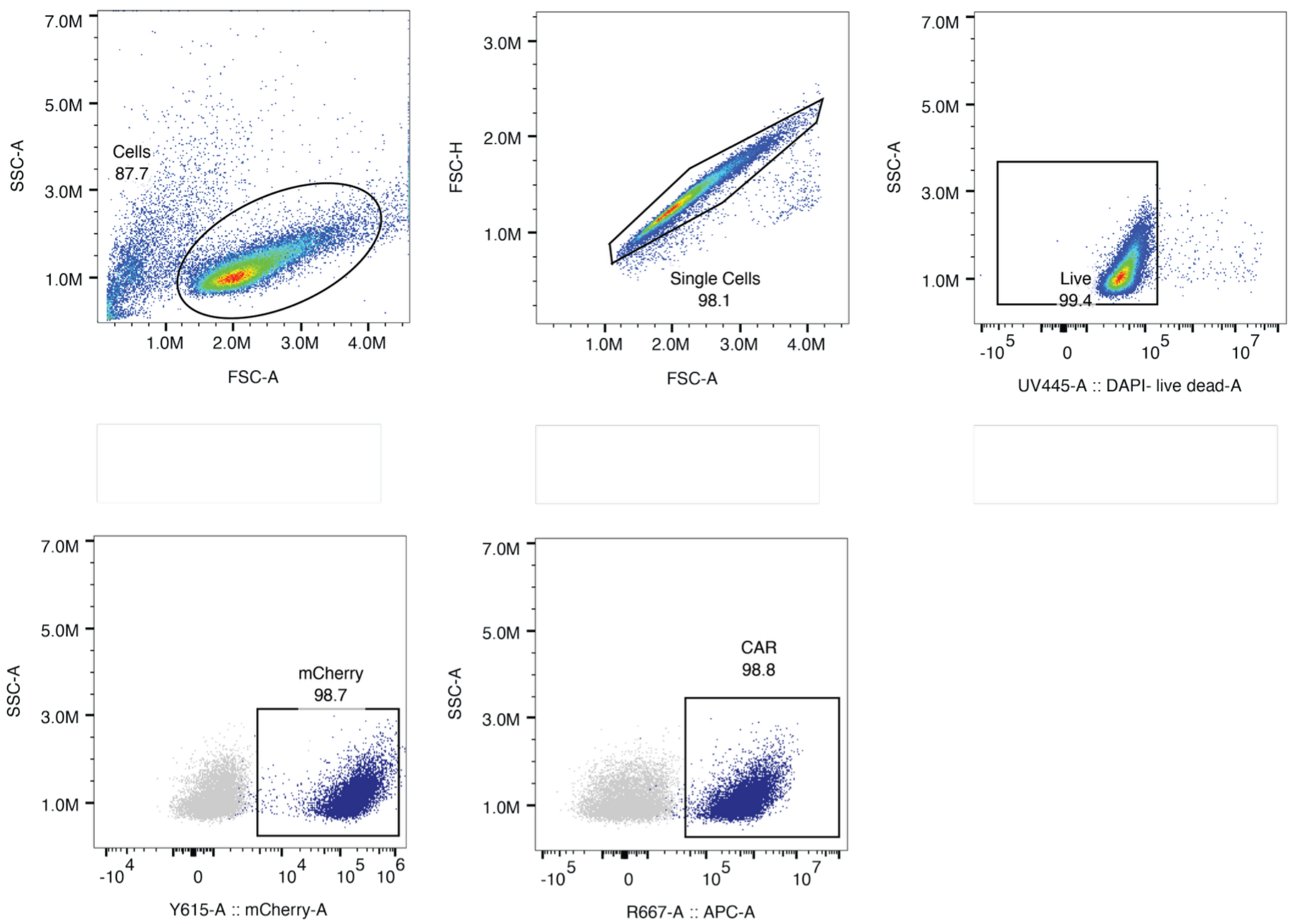

Figure S1. Gating strategy for CAR-T cells at end of production. Sequential gating on cells (FSC-A/SSC-A), single cells (FSC-A/FSC-H), live cells (DAPI⁻), mCherry⁺, and CAR⁺ (BCMA-APC) populations. Representative plots from one donor.

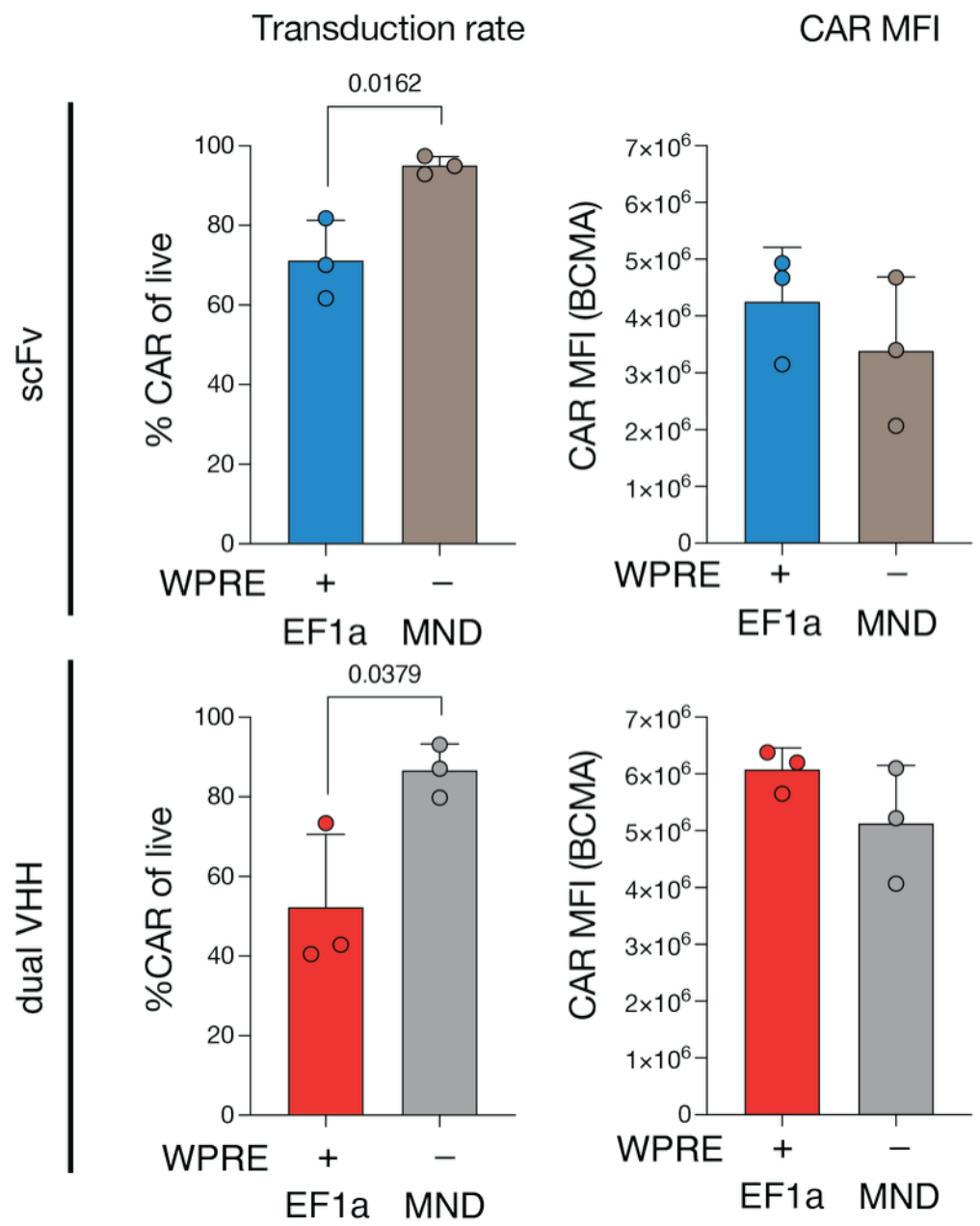

Figure S2. Transduction rates and CAR surface MFI of NFAT-GFP Jurkat reporter cells.

Transduction rate (%CAR⁺ of live) and CAR surface MFI (BCMA-APC) for EF1α+WPRE versus MND-WPRE, scFv and dual VHH, confirming matched CAR expression used for the tonic-signaling comparison in figure 2. Data are mean±SEM of n=3 replicates. P-values of unpaired t-tests are shown.

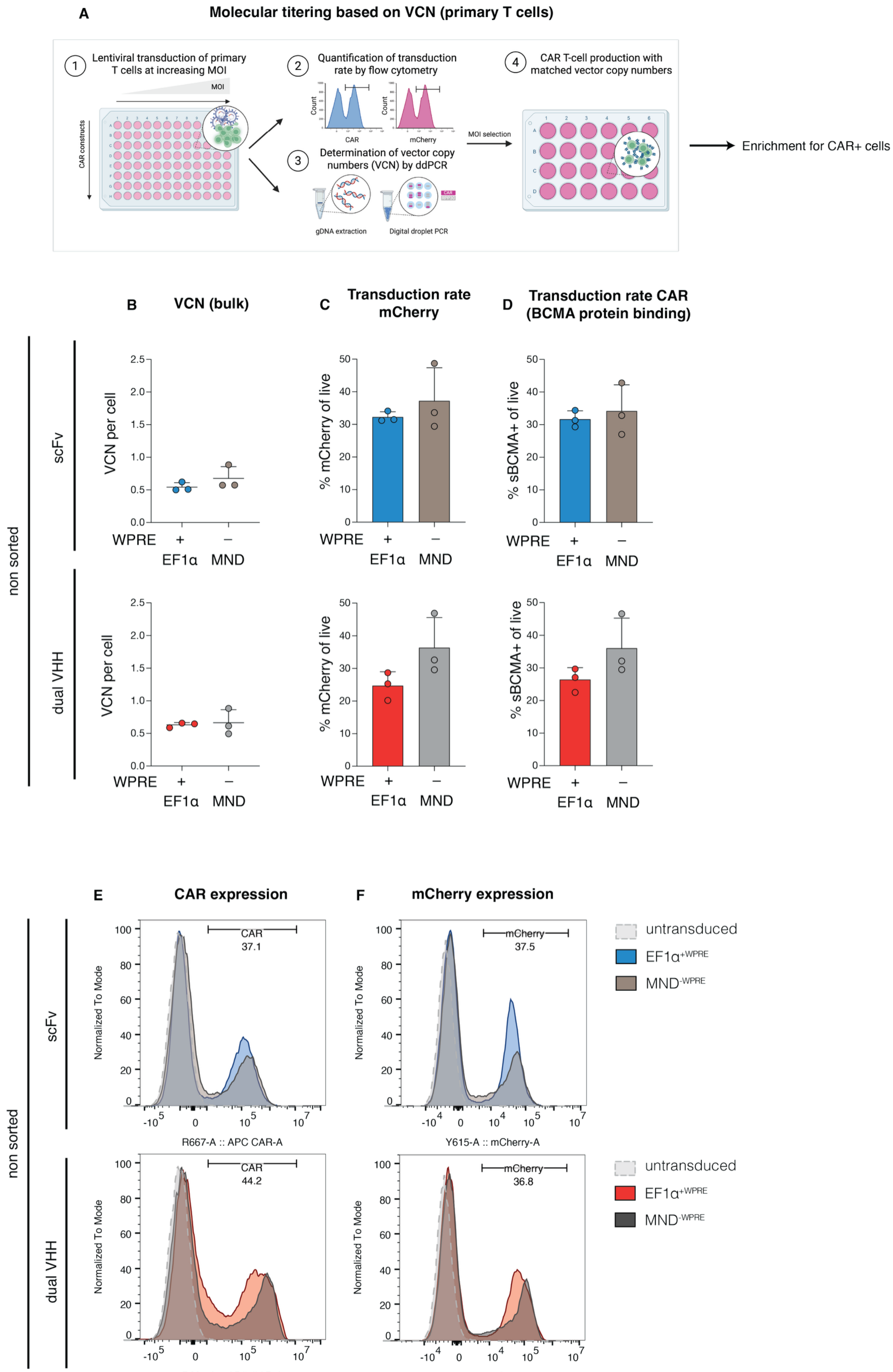
A
Molecular titering based on VCN (primary T cells)
1 Lentiviral transduction of primary T cells at increasing MOI
MOI
CAR constructs
2 Quantification of transduction rate by flow cytometry
Count
CAR
mCherry
3 Determination of vector copy numbers (VCN) by ddPCR
gDNA extraction
Digital droplet PCR
MOI selection
4 CAR T-cell production with matched vector copy numbers
Enrichment for CAR+ cells
B
VCN (bulk)
C
Transduction rate mCherry
D
Transduction rate CAR (BCMA protein binding)
non sorted
scFv
dual VHH
VCN per cell
% mCherry of live
% sBCMA+ of live
WPRE
+
–
EF1α
MND
E
CAR expression
F
mCherry expression
CAR
37.1
44.2
mCherry
37.5
36.8
Normalized To Mode
R667-A :: APC CAR-A
Y615-A :: mCherry-A
untransduced
EF1α+WPRE
MND-WPRE

Figure S3. Titering of lentiviral vectors and transgene expression in unsorted bulk population.

(A) Workflow for molecular titration to matched VCN in primary T cells: transduction across an MOI series, quantification of transduction rate by flow cytometry (CAR, mCherry), VCN determination by ddPCR, and selection of MOI yielding matched VCN for CAR-T cell production. (B) VCN per cell in the bulk population. (C) Transduction rate as %mCherry$^+$ of live cells. (D) Transduction rate as %CAR$^+$ of live cells. Mean±SEM. (E-F) Representative histograms of CAR (BCMA-APC, E) and mCherry expression (F) for scFv and dual VHH constructs, percentage positive annotated. UTD, untransduced. n=3 biological replicates from three independent human T-cell donors.

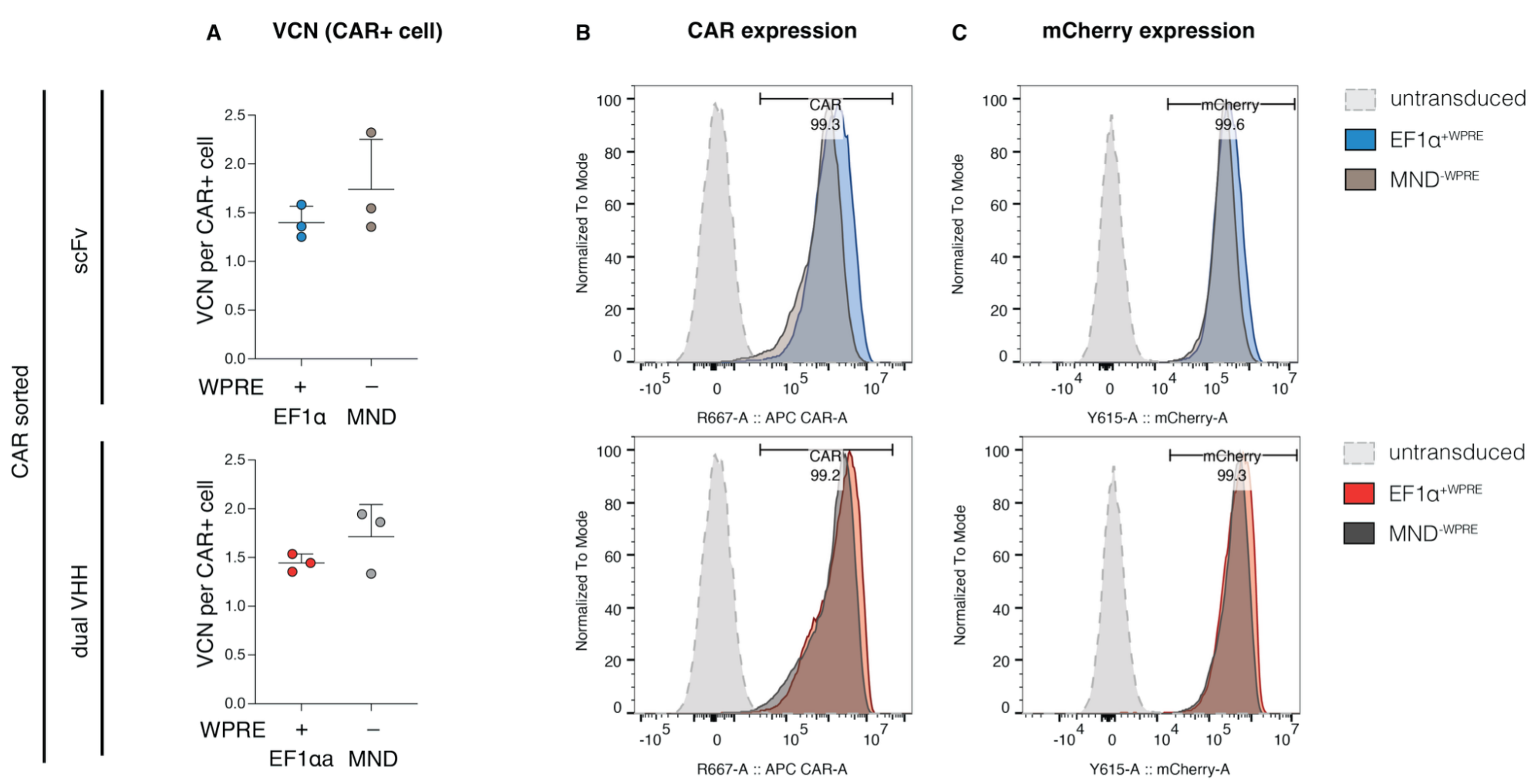

Figure S4. VCN, CAR and mCherry expression after CAR enrichment.

(A) VCN per CAR$^+$ cell. (B-C) Representative histograms of CAR (BCMA-APC, B) and mCherry expression (C) for scFv and dual VHH constructs, percentage positive annotated. UTD, untransduced. n=3 biological replicates from three independent human T-cell donors.
